# Effect of Drought on the Methylerythritol 4-Phosphate (MEP) Pathway in the Isoprene Emitting Conifer *Picea glauca*

**DOI:** 10.3389/fpls.2020.546295

**Published:** 2020-10-09

**Authors:** Erica Perreca, Johann Rohwer, Diego González-Cabanelas, Francesco Loreto, Axel Schmidt, Jonathan Gershenzon, Louwrance Peter Wright

**Affiliations:** ^1^Department of Biochemistry, Max Planck Institute for Chemical Ecology, Jena, Germany; ^2^Laboratory for Molecular Systems Biology, Department of Biochemistry, Stellenbosch University, Stellenbosch, South Africa; ^3^Consiglio Nazionale delle Ricerche, Dipartimento di Scienze Bio-Agroalimentari, Roma, Italy

**Keywords:** carotenoid, chlorophyll, DXS enzyme, metabolic flux, alternative carbon source, MVA pathway

## Abstract

The methylerythritol 4-phosphate (MEP) pathway of isoprenoid biosynthesis produces chlorophyll side chains and compounds that function in resistance to abiotic stresses, including carotenoids, and isoprene. Thus we investigated the effects of moderate and severe drought on MEP pathway function in the conifer *Picea glauca*, a boreal species at risk under global warming trends. Although moderate drought treatment reduced the photosynthetic rate by over 70%, metabolic flux through the MEP pathway was reduced by only 37%. The activity of the putative rate-limiting step, 1-deoxy-D-xylulose-5-phosphate synthase (DXS), was also reduced by about 50%, supporting the key role of this enzyme in regulating pathway metabolic flux. However, under severe drought, as flux declined below detectable levels, DXS activity showed no significant decrease, indicating a much-reduced role in controlling flux under these conditions. Both MEP pathway intermediates and the MEP pathway product isoprene incorporate administered ^13^CO_2_ to high levels (75–85%) under well-watered control conditions indicating a close connection to photosynthesis. However, this incorporation declined precipitously under drought, demonstrating exploitation of alternative carbon sources. Despite the reductions in MEP pathway flux and intermediate pools, there was no detectable decline in most major MEP pathway products under drought (except for violaxanthin under moderate and severe stress and isoprene under severe stress) suggesting that the pathway is somehow buffered against this stress. The resilience of the MEP pathway under drought may be a consequence of the importance of the metabolites formed under these conditions.

## Introduction

Nearly all members of the vast isoprenoid family of metabolites are produced from the two C_5_ diphosphate intermediates, dimethylallyl diphosphate (DMADP) and its isomer isopentenyl diphosphate (IDP). These intermediates arise from two different pathways in plants, the mevalonate (MVA) pathway located in the cytosol, ER and peroxisomes, and the more recently identified methylerythritol 4-phosphate (MEP) pathway localized in plastids (Hemmerlin et al., 2012). While C_5_ units derived from the MVA pathway are used in the formation of compounds such as sesquiterpenes, sterols, brassinosteroids, triterpenes, dolichols and farnesylated proteins, C_5_ units from the MEP pathway are used in the production of isoprene, monoterpenes, diterpenes, chlorophylls, and carotenoids as well as the gibberellin, strigolactone and abscisic acid hormones. The extent to which each of the two pathways contributes to the total DMADP/IDP pool under various conditions has not been completely elucidated. Eberl et al. (2018) suggested a contribution to the total DMADP/IDP intermediate pool size from the MVA pathway, under fungus infestation in poplar leaves. Dudareva et al. (2005) showed that the MEP pathway provides IDP precursors for both plastidial monoterpene and cytosolic sesquiterpenes synthesis in snapdragon flowers and pointed out the possibility of a cross talk between the two pathways. However, other studies showed that cross talk between both pathways is not capable of rescuing a pharmacological block in either pathway (Laule et al., 2003; Rodríguez-Concepción et al., 2004).

Isoprenoids have a wide variety of functions in plant growth, development, reproduction and defense. Among the MEP pathway products, several classes protect against oxidative stress, including carotenoids, tocopherols and isoprene. The formation of these compounds might be favored under conditions leading to oxidative stress, including high temperature, high light and low water supply. Experiments with high light and high temperature actually point to the possibility that metabolic flux through the MEP pathway is reduced in a number of plant species because of the inhibition of the two ultimate steps catalyzed by proteins with oxygen-sensitive 4Fe-4S clusters, leading to the accumulation of the intermediate 2-*C*-methyl-D-erythritol-2,4-cyclodiphosphate (MEcDP) (Rivasseau et al., 2009). However, little work has been done on the effects of drought in this context, and no comprehensive study has been performed on the MEP pathway in a conifer species. The effect of drought on plant metabolism in general has been studied for many years, but most attention has been focused on the increased synthesis of various osmolytes, such as quaternary ammonium compounds and polyhydric alcohols. There are also scattered reports on alterations in other pathways of primary and secondary metabolism (Selmar, 2008; Guo et al., 2018; Mundim and Pringle, 2018; Ahkami et al., 2019). Among isoprenoids, phytol and α-tocopherol in *Brachypodium distachyon* were reported to increase during early phases of drought vs. well-watered control plants, but declined during later drought phases (Ahkami et al., 2019). Meanwhile, secondary metabolite isoprenoids were shown in a meta-analysis to generally increase during drought (Mundim and Pringle, 2018), but the systematic investigation of drought effects on an isoprenoid pathway has not been undertaken.

Control of the MEP pathway is manifested at several different levels of organization. The first enzymatic step, 1-deoxy-D-xylulose-5-phosphate synthase (DXS), has been generally assumed to be rate limiting based on studies in which overexpression of the corresponding gene led to increases in MEP pathway products (Estévez et al., 2001; Enfissi et al., 2005). In *Arabidopsis thaliana*, DXS was shown to control approximately 80% of the metabolic flux through the MEP pathway in photosynthetic tissue by metabolic control analysis (Wright et al., 2014). DXS activity itself is regulated transcriptionally and post-transcriptionally (Rodríguez-Concepción, 2006; Banerjee and Sharkey, 2014). In particular, feedback inhibition of DMADP and IDP regulates DXS activity in some species (Banerjee et al., 2013). Other factors regulating the MEP pathway include the supply of the two initial substrates, glyceraldehyde-3-phosphate and pyruvate (Banerjee and Sharkey, 2014). Moreover, under certain conditions the intermediate MEcDP is subject to efflux from the pathway (Wright et al., 2014; González-Cabanelas et al., 2015), and appears to be the source of a plastid-to-nucleus signal that regulates salicylic acid signaling (Xiao et al., 2012; Onkokesung et al., 2019). Regulation of the MEP pathway has been well studied in angiosperms (Banerjee et al., 2013; Ghirardo et al., 2014), whereas information about gymnosperms, especially conifers, is very limited, especially under drought. Here we investigate the effect of drought on the MEP pathway in the widespread boreal conifer *Picea glauca*, one of the few conifers that emit both isoprene and monoterpenes (Loreto and Fineschi, 2015). As boreal forests are increasingly affected by global warming (Soja et al., 2007; Price et al., 2013), this study may help understand the consequences of drought on tree metabolism in a critical ecosystem.

We found drought to decrease metabolic flux through the MEP pathway in *P. glauca*, although this decline was considerably less than the declines in photosynthesis and transpiration observed, and there was no apparent decrease in the levels of most major MEP pathway products. Under moderate drought, decrease in pathway flux seemed to be modulated by the activity of DXS, but this enzyme had little contribution to the regulation of the MEP pathway under severe drought.

## Materials and Methods

### Plant Material and Drought Treatment

Young trees of white spruce [*Picea glauca* (Moench) Voss], 3 years old, were purchased from a local nursery in Jena, Germany and grown outdoors at the Max Planck Institute of Chemical Ecology under natural conditions. At the end of May 2015, the trees were transferred inside a greenhouse with supplemental lighting. Relative humidity was maintained between 50 and 60%, light period was 14 h, and the temperature was 23°C:19°C, day: night. The experiment was performed in August after needles had fully expanded. Drought was applied by withholding water. At the beginning of the experiment, trees were irrigated and excess of water was allowed to drain for 2–3 h. Then the initial pot weight was measured using a digital balance to a precision of 1 g (model QS32A; Sartorius Instrumentation, Göttingen, Germany). Subsequently, pot weights were recorded daily. Each pot was enclosed with a bag to avoid soil evaporation. Therefore, loss of water was attributed to plant transpiration only. The experiment lasted 20 days. The availability of water under the different stress regimes, was described by the fraction of transpirable soil water (FTSW). The FTSW was calculated by using the daily pot weight according to the formula (Sinclair and Ludlow, 1986; Ray and Sinclair, 1998):

FTSW=(daily⁢weight-final⁢weight)/(initial⁢weight-final⁢weight)

The final pot weight was reached when soil water content no longer supports transpiration and corresponded to the FTSW endpoint (Sinclair and Ludlow, 1986). The relative transpiration rate (RTR) of the trees was calculated by their daily transpiration rate (TR), determined by daily pot weight loss, divided by the average transpiration rate (ATR) of a group of well-watered trees (Sinclair and Ludlow, 1986; Ray and Sinclair, 1998):

RTR=(TR/ATR)×100

Over the course of the experiment, these well-watered trees were irrigated daily until pot capacity and weighed. They were not used in any further measurements. Three treatments were performed: control, moderate drought and severe drought. We used five trees for each treatment, and the treatments were sampled at different times. The control trees were watered daily and sampled only at the end of the experiment. Stressed trees, from which water was completely withheld, were sampled when the target RTR (50% for moderate stress at FTSW12, 20% for severe stress at FTSW3) was achieved (Supplementary Figure S1). Sampling consisted of measuring from one twig the photosynthetic rate and isoprene emission, and labeling with ^13^CO_2_ for 50 min. Afterward the same twig was harvested for metabolite analysis. This twig was always the youngest branch on the shoot, and had flushed in the current year. After sampling, trees were allowed to dry further until RTR was 10%, corresponding to the FTSW endpoint (Sinclair and Ludlow, 1986).

### Sample Processing

After ^13^CO_2_ labeling for 50 min (see details below), twigs were harvested, frozen immediately in liquid nitrogen, and transferred to a −80°C freezer. Only needles were used for biochemical analysis. After grinding, the total fresh weight was measured. Analysis of chlorophylls, carotenoids, β-cyclocitral, monoterpenes, and the DXS assay was carried out with fresh tissue, while analyses of MEP intermediate metabolites, sugars, and abscisic acid were carried out after freeze-drying. For each tree, 100 mg of fresh tissue were weighed before and after freeze-drying to determine fresh-to-dry weight conversion factors. Due to the differences in leaf water content between stressed and control trees all measurements were reported on a dry weight basis. Rates of photosynthesis and isoprene emission were also referenced to the dry weight of the needles.

### Photosynthetic Rate Measurement

Photosynthesis was measured with a portable gas exchange system (LI-6400; LI-COR, Lincoln, NE, United States) using a chamber for measuring conifer needles supplied with the instrument. Measurements were performed between 10:00 and 14:00 under conditions of photosynthetic photon flux density (PPFD) (1000 μmol m^–2^ s^–1^). Leaf temperature was set at 30°C, and the relative humidity in the cuvette ranged between 45 and 50%. After harvesting the measured tissue, *A*, the rate of carbon fixation, was calculated on a dry weight basis according to the LI-COR manual pages 16–51.

### Abscisic Acid (ABA) Analysis

A 10 mg quantity of freeze-dried, ground leaf material was extracted with 1 ml methanol containing 40 ng ml^–1^ D_6_-abscisic acid (Santa Cruz Biotechnology, Dallas, TX, United States) as an internal standard. The solution was incubated at 20°C for 30 min in a heating block shaking at 1000 rpm. After centrifugation at 18,000 × *g* at 4°C for 20 min, the supernatant was analyzed by using an Agilent 1260 Infinity high-performance liquid chromatography (HPLC) system (Agilent, Santa Clara, CA, United States) coupled to an API 5000 tandem mass spectrometer (AB Sciex, Framingham, MA, United States). A Zorbax Eclipse XDB-C18 column (50 × 4.6 mm, 1.8 μm) was used for the chromatographic separation with a formic acid (0.05% in water)/acetonitrile gradient (flow, 1.1 ml min^–1^). ABA was detected via multiple reaction monitoring and quantified relative to the peak area of the standard.

### Sugar Analysis

Freeze dried needles (5 mg) were extracted with 1 ml 80% MeOH. The solution was vortexed for 10 min at ambient temperature and centrifuged at 13,000 × *g* and 4°C for 5 min. The extracts were further diluted 1:10 with water before analysis on an Agilent 1260 Infinity HPLC system connected to an API 5000 triple quadrupole mass spectrometer. An external standard curve made with authentic standards (Sigma-Aldrich) at concentrations ranging between 1.25 and 20.0 μg ml^–1^ was used for quantification. Samples were analyzed directly by LC-MS/MS after a 1:20 (v/v) dilution in water. Separation was made via hydrophilic interaction chromatography on an apHera NH_2_ polymer column (5 μm, 15 × 4.6 mm, Supelco, Bellefonte, PA, United States). Water and acetonitrile were used as mobile phases A and B, respectively. The elution profile was: 0–0.5 min, 80% B in A; 0.5–13 min, 80–55% B in A; 13–14 min, 55–80% B in A; and 14–18 min, 80% B in A. The ion spray voltage was maintained at −4500 eV. The turbo gas temperature was set at 600°C. Nebulizing gas was set at 50 psi, curtain gas at 20 psi, heating gas at 60 psi, and collision gas at 5 psi. Multiple reaction monitoring (MRM) was used to monitor isotopic composition of glucose and fructose by the following precursor ion → product ion reactions: *m*/*z* 178.8 → 58.7, *m*/*z* 179.8 → 58.7, *m*/*z* 179.8 → 59.7, *m*/*z* 180.8 → 58.7 *m*/*z* 180.8 → 59.7, and *m*/*z* 180.8 → 60.70. For sucrose, we used *m*/*z* 340.9 → 58.8, *m*/*z* 341.9 → 58.8, *m*/*z* 341.9 → 59.8, *m*/*z* 342.9 → 58.8 *m*/*z* 342.9 → 59.8, *m*/*z* 342.9 → 60.8, *m*/*z* 343.9 → 58.8, *m*/*z* 343.9 → 59.8, *m*/*z* 343.9 → 60.8, *m*/*z* 344.9 → 58.8, *m*/*z* 344 → 59.8, *m*/*z* 344.9 → 60.8, *m*/*z* 345.9 → 58.8, *m*/*z* 345.9 → 59.8, *m*/*z* 345.9 → 60.8, *m*/*z* 346.9 → 58.8, *m*/*z* 346.9 → 59.8, *m*/*z* 346.9 → 60.8, *m*/*z* 347.9 → 58.8, *m*/*z* 347.9 → 59.8, *m*/*z* 347.9 → 60.8, *m*/*z* 348.9 → 58.8, *m*/*z* 348.9 → 59.8, *m*/*z* 348.9 → 60.8, *m*/*z* 349.9 → 58.8, *m*/*z* 349.9 → 59.8, *m*/*z* 349.9 → 60.8, *m*/*z* 350.9 → 58.8, *m*/*z* 350.9 → 59.8, *m*/z 350.9 → 60.8, *m*/*z* 351.9 → 59.8, *m*/*z* 351.9 → 60.8, and *m*/*z* 352.9 → 60.8. The percentage of ^13^C labeling in each metabolite after 50 min was calculated by summing all ^13^C atoms incorporated in the sugar isotopes, and dividing this number by the overall sum of unlabeled and labeled C atoms.

### Analysis of Methylerythritol 4-Phosphate (MEP) Pathway Intermediate Metabolites and ^13^C-Labeling

To quantify the MEP pathway metabolites, 1-deoxy-D-xylulose 5-phosphate (DXP), 2-C-methyl-D-erythritol 4-phosphate (MEP), 4-diphosphocytidyl-2-C-methyl-D-erythritol (CDP-ME), 2-C-methyl-D-erythritol-2,4-cyclodiphosphate (MEcDP), isopentenyl diphosphate and dimethylallyl diphosphate (IDP + DMADP), samples of 5 mg dry weight were extracted twice with 250 μl of 50% acetonitrile containing 10 mM ammonium acetate, pH 9.0. After vortexing and centrifugation in a micro-centrifuge at 20,000 × *g* for 5 min, 200 μl of the supernatant from both extracts were combined, transferred into a new 1.5 ml tube and dried under a stream of nitrogen gas at 40°C. The residue was dissolved in 100 μl of 10 mM ammonium acetate, pH 9.0, and after vortexing, 100 μl of chloroform was added. The upper aqueous phase, separated by centrifugation at 20,000 × *g*, was transferred into a new tube and diluted with 1 volume of acetonitrile. To remove any precipitate, the supernatant was transferred to an HPLC vial after centrifugation at 20,000 × *g*.

Methylerythritol 4-phosphate pathway metabolites were analyzed on an Agilent 1260 Infinity HPLC system connected to an API 5000 triple quadrupole mass spectrometer. A 5 μl quantity of the extract was injected and the metabolites were separated on a hydrophobic interaction liquid chromatography (HILIC) XBridge Amide columns (150 × 2.1 mm, 3.5 μm; Waters, Milford, MA, United States) with a HILIC guard column containing the same sorbent (10 × 2.1 mm, 3.5 μm) and a SSI^TM^ high pressure pre-column filter (Sigma-Aldrich, St. Louis, MI, United States) using two solvents: 20 mM ammonium bicarbonate adjusted to pH 10.5 with LC-MS grade ammonium hydroxide (solvent A) and 80% acetonitrile containing 20 mM ammonium bicarbonate, pH 10.5 (solvent B). The solvent gradient profile started with 100% of solvent B, which decreased to 60% in the first 15 min, followed by an isocratic elution with 100% solvent B. Separation was performed at 25°C with a flow rate of 500 μl min^–1^.

The mass spectrometer was used in the negative ionization mode with the following instrument settings: ion spray voltage −4500 V, turbo gas temperature 700°C, nebulizer gas 70 psi, heating gas 30 psi, curtain gas 30 psi, and collision gas 10 psi. DXP and its isotope distribution were monitored by the following precursor ion → product ion reactions: *m*/*z* 212.9 → 96.9, *m*/*z* 213.9 → 96.9, *m*/*z* 214.9 → 96.9, *m*/*z* 215.9 → 96.9, *m*/*z* 216.9 → 96.9, and *m*/*z* 217.9 → 96.9 [collision energy (CE), −16 V; declustering potential (DP), −60 V; and cell exit potential (CXP), −15 V]. MEcDP and its isotope distribution were monitored by the following precursor ion → product ion reactions: *m*/*z* 277.0 → 78.9, *m*/*z* 278.0 → 78.9, *m*/*z* 279.0 → 78.9, *m*/*z* 280.0 → 78.9, *m*/*z* 281.0 →7 8.9, and *m*/*z* 282.0 → 78.9 (CE, −38 V; DP, −50 V; CXP, −11 V). IDP/DMADP and their isotope distributions were monitored by the following precursor ion → product ion reactions: *m*/*z* 244.9 → 78.9, *m*/*z* 245.9 → 78.9, *m*/*z* 246.9 → 78.9, *m*/*z* 247.9 → 78.9, *m*/*z* 248.9 → 78.9, and *m*/*z* 249.9 → 78.9 (CE, −24 V; DP, −45 V; CXP, −11 V). Analyst 1.6 software (Applied Biosystems) was used for data acquisition and processing.

The metabolite concentrations were quantified by using external standard curves and were normalized to unlabeled standards added to each extract, after correction for natural ^13^C abundance. Normalization to added unlabeled standards was accomplished by analyzing each sample twice, once with and once without the addition of 25 ng of DXP, 55 ng of MEcDP, and 24 ng of DMADP + IDP standards dissolved in 10 ml of water (Wright et al., 2014). Percentage of ^13^C labeling in each metabolite after 50 min was calculated by summing all ^13^C atoms incorporated in the various isotopologues, and dividing this number by the overall sum of unlabeled and labeled C atoms.

### Measurement of Isoprene Emission and Incorporation of ^13^CO_2_

Isoprene emission was analyzed when needles were in the cuvette for photosynthesis measurements. A proton transfer reaction mass spectrometer (PTR-MS; Ionicon, Innsbruck, Austria) (Lindinger et al., 1998) was employed with a Gas Calibration Unit (Ionicon) to generate precise flows of an isoprene standard for calibration. The PTR-MS was attached to the outflow of the LI-COR 6400 cuvette. The drift tube pressure was 2.2–2.3 mbar and the E/N ratio (electric field/particle density) was 130 Td (1 Td = 1 Townsend = 10^–17^ cm^2^ V^–1^ s^–1^). Isoprene was monitored with the mass signal *m*/*z* 69. The raw count-rate (cps) of the isoprene signal was normalized (ncps) to the sum of the primary ion and water cluster, and to the drift tube pressure. The average of the normalized signal during the steady state period was used to calculate the emission rate, after subtracting the background (empty chamber without needles). *In vivo* labeling was accomplished by replacing the ^12^CO_2_ in the air entering the cuvette with ^13^CO_2_ (0.99 atom% ^13^C; Linde) at the same ambient concentration (380 μmol mol^–1^). Labeling was performed for 50 min. The appearance of protonated masses of isoprene was followed in the PTR MS by monitoring *m*/*z* 70 (^13^C^12^C_4_H_9_), *m*/*z* 71 (^13^C_2_^12^C_3_H_9_), *m*/*z* 72 (^13^C_3_^12^C_2_H_9_), *m*/*z* 73 (^13^C_4_^12^CH_9_), and *m*/*z* 74 (^13^C_5_H_9_). The percentage of ^13^C labeling was calculated by summing all ^13^C atoms incorporated in the isoprene isotopes, and dividing this number by the overall sum of unlabeled and labeled carbon atoms of isoprene (Schnitzler et al., 2004). Isoprene emission was normalized to the dry weight of the needles.

### Preparation of Soluble Protein Extract and Measurement of DXS Activity

Freshly ground needles (200 mg) were immersed in 1 ml of extraction buffer containing 250 mM MOPSO (pH 6.8), 5 mM ascorbic acid, 5 mM sodium bisulfite, 5 mM dithiothreitol (DTT), 10 mM MgCl_2_, 1 mM ethylenediaminetetraacetic acid (EDTA), 10% (v/v) glycerol, 1% (w/v) polyvinylpyrrolidone (PVP, M_r_ = 10,000), 4% (w/v) polyvinylpolypyrrolidone (PVPP), 4% (w/v) Amberlite XAD-4, and 0.1% (v/v) Tween 20. Extraction was carried out in an 1.5 ml Eppendorf tube shaken in an Eppendorf Thermoshaker at 4°C and 1400 rpm. After centrifugation for 20 min at 4°C and 20,000 × *g*, the supernatant was passed through a 2 ml Zeba Spin desalting column with a 7 kDa molecular weight cut-off (Thermo Scientific, Rockford, IL, United States) to exchange the buffer to 50 mM Tris–HCl, pH 8.0, with 10% (v/v) glycerol and 10 mM MgCl_2_. The total protein concentration was estimated by measuring absorbance at 280 nm using a NanoDrop 1000 UV-Vis spectrophotometer (Thermo Scientific).

The DXS enzyme assay was carried out in accord with a previous protocol (Wright and Phillips, 2014). Briefly, 30 μl from the total volume of enzyme extract was combined with 70 μl of assay buffer of 50 mM Tris–HCl, pH 8.0, with 10% (v/v) glycerol, 10 mM MgCl_2_, 2.5 mM dithiothreitol, 1 mM thiamine pyrophosphate, 2 mM imidazole, 1 mM sodium fluoride, 1.15 mM molybdate, 1% (v/v) protease inhibitor cocktail, and 10 mM each of the substrates pyruvate and glyceraldehyde-3-phosphate. The final volume of 100 μl was incubated in a water bath at 25°C for 2 h. As a control for non-enzymatic conversion and the presence of assay product in the original plant extract, 30 μl of the enzyme extract were heated to 90°C for 10 min to deactivate the enzyme, and then combined with 70 μl of mixture assay and incubated for 2 h as well. The enzyme reaction was stopped by vigorously vortexing for 5 s with 100 μl of chloroform. After centrifugation in a microcentrifuge to complete phase separation, the upper aqueous phase, was transferred into a new tube and diluted with 1 volume of acetonitrile. As an internal standard, 25 ng of [^13^C_5_] DXP dissolved in water was added to the final solution dissolved in water.

The enzymatic end product DXP was quantified on an Agilent 1260 Infinity HPLC system connected to an API 5000 triple quadrupole mass spectrometer. DXP was separated via hydrophilic interaction liquid chromatography with the column and solvent system mentioned above for the analysis of MEP pathway metabolites. The flow rate was 1 ml min^–1^ with a column temperature of 25°C. The solvent profile started with a linear gradient from 0 to 16% A over 5 min and followed with an isocratic separation for 10 min. After a linear gradient from 16 to 40% A over 5 min, solvent A was returned to 0% over 15 min of equilibration. The mass spectrometer was used in the negative ionization mode with the following instrument settings: ion spray voltage −4500 V, turbo gas temperature 700°C, nebulizer gas 70 psi, heating gas 30 psi, curtain gas 30 psi, and collision gas 10 psi. DXP was monitored using the following precursor ion → product ion transition *m*/*z* 212.95 → 78.9 and DXP. The DXP produced by the DXS enzyme reaction was normalized to the [^13^C_5_] DXP internal standard monitored with the transition *m*/*z* 217.94 → 96.9.

### Metabolic Flux Calculations

Plastidial concentrations of DXP, MEcDP and IDP + DMADP were estimated by assuming that IDP + DMADP only occurred in the chloroplast, and that only plastidial DXP and MEcDP pools would be labeled on the time-scale of the labeling experiment (50 min). Thus, the plastidial concentrations of DXP and MEcDP were estimated by calculating the ratio of their final ^13^C-label incorporation to that of IDP + DMADP, and using these fractions to determine plastidial content. As isoprene labeling could be followed on-line instantaneously with the PTR-MS without the need for individual sampling, these measurements were taken as the instantaneous labeling state of the IDP + DMADP pool. This assumption was justified since isoprene is produced from DMADP in a single step, and the volatile isoprene gas escapes from the leaf. Moreover the assumption was verified experimentally by ensuring that the final label incorporation after 50 min in the isoprene and IDP + DMADP pools was identical (Figure 5). Following an approach similar to Yuan et al. (2008), the differential equations for label incorporation were integrated to obtain an analytical expression for the fractional labeling of the IDP + DMADP pool with time as a function of the pool sizes of DXP, MEcDP, and IDP + DMADP, as well as the flux through the pathway:

(1) $$\begin{array}{r} f\left(t\right)=m\left\{1-\frac{A^{2}}{\left(A-B\right)\left(A-C\right)}\exp\left(-\frac{J}{A}t\right)\right.\\ 1.2pc-\frac{B^{2}}{\left(B-A\right)\left(B-C\right)}\exp\left(-\frac{J}{B}t\right)\\ 1.2pc\left.-\frac{C^{2}}{\left(C-A\right)\left(C-B\right)}\exp\left(-\frac{J}{C}t\right)\right\} \end{array}$$

where *f(t)* is the fractional labeling of isoprene (which equates to the fractional labeling of IDP + DMADP) as a function of time, *m* is the maximal fractional labeling at the end of the run, *A, B*, and *C* are the pool sizes of DXP, MEcDP, and IDP + DMADP, respectively, *J* is the pathway flux and *t* is time. Equation (1) assumes that the pools of the other MEP pathway intermediates (MEP, ME-CDP, MEP-CDP, and HMBDP) are too small to significantly delay the label incorporation into downstream metabolites; these intermediates were below the limit of detection in the HPLC-MS analysis. To calculate the flux, Eq. (1) was fitted to isoprene labeling time-courses obtained from the PTR-MS [*f*(*t*)], with the plastidial pool sizes of DXP, MEcDP and IDP + DMADP entered as parameters *A*, *B*, and *C*, respectively (Supplementary Figure S2). The parameters *m* and *J* were obtained by minimizing the sum of squares of the differences between model and data with the Levenberg-Marquardt algorithm as implemented in the Python LMFIT module (Newville et al., 2014).

### Chlorophyll and Carotenoid Analysis

After grinding the needles in liquid nitrogen, 50 mg were extracted in light-protected tubes with 1 ml of acetone by shaking for 6 h at 4°C in the dark. After centrifugation for 5 min at 2300 × *g* and 4°C, 800 μl of the extract was transferred into a light-protected tube and 200 μl of water was added. After centrifuging the samples for 1 min at 2300 × *g* and 4°C, they were transferred to brown glass vials for analysis on an HPLC Agilent 1100 Series with UV diode-array-detector. The detector was set at 445 nm for carotenoids and at 650 nm for the chlorophylls. These pigments were separated on a Supelcosil column LC-18 (7.5 cm × 4.6 mm, 3 μm; Sigma Aldrich) by using a gradient of acetone (solvent A) and 1 mM NaHCO_3_ in water (solvent B). The flow rate was 1.5 ml min^–1^. The initial mobile phase consisted of 65/35% (v/v) solvent A/solvent B. Then, solvent A was linearly increased up to 90% in 12 min and to 100% in 8 min. 100% solvent A was kept for 2 min and then decreased to 65% in 3 min. Quantification was done using external standard curves. Authentic standards of chlorophylls and β-carotene (Santa Cruz Biotechnology) were analyzed in a range from 0.1 to 0.00625 mg ml^–1^. Lutein, neoxanthin, and violaxanthin were considered to have the same response factors as β-carotene.

### β-Cyclocitral Analysis

A 100 mg quantity of fresh tissue was extracted with 1 ml of methanol. The suspension was mixed by vortexing for 5 min and centrifuged for 20 min at 20, 000 × *g* and 4°C. The supernatant was centrifuged as above for 10 min, and 200 μl of the new supernatant were taken for the analysis using an Agilent 1260 Infinity high-performance liquid chromatography (HPLC) system coupled to an API 5000 tandem mass spectrometer. β-Cyclocitral was separated on an Zorbax Eclipse XDB-C18 column (50 × 4.6 mm, 1.8 μm) Chromatographic separation was performed by using a gradient of formic acid 0.05% in water, (solvent A) and acetonitrile (solvent B). The flow rate was set at 0.5 ml min^–1^. The initial mobile phase consisted of 95/5% (v/v) solvent A/solvent B. Then, solvent A was decreased to 50% in 2 min and held for 3 min. After solvent A was decreased to 0% in 9 min and held for 11 min, it was raised again to 95%. β-Cyclocitral was monitored by following the precursor ion → product ion reaction: *m*/*z* 153.191 → 109.0. Quantification was done using an external standard curve made with an authentic standard of β-cyclocitral (Sigma-Aldrich).

### Monoterpene Content Analysis

According to the procedure of Martin et al., 2002 fresh needles (100 mg) were immersed in 1.5 ml of tert-butyl methyl ether containing 150 μg ml^–1^ isobutylbenzene as internal standard, and shaken for 14 h at room temperature. The ethereal extract was transferred to a fresh vial and washed with 0.3 ml of 0.1 M (NH_4_)_2_CO_3_ (pH 8.0) in order to purify the extracted terpenes from other organic acids. The sample was filtered through a Pasteur pipette column filled with silica gel (Sigma 60 Å) and anhydrous MgSO_4_. Monoterpene analysis was performed by GC-MS with a Hewlett-Packard 6890 system, using a DB-WAX column (0.25 mm × 30 m, 0.25 μm, J&W Scientific, Folsom, CA, United States). Split injection was carried out at 220°C. Helium was used as carrier gas at a constant flow of 1 ml min^–1^. The GC was programmed with an initial oven temperature of 40°C (3-minute hold), a ramp of 5°C min^–1^ until 80°C, then a ramp of 5°C min^–1^ until 200°C, followed by a final ramp of 60°C min^–1^ until 280°C (4-minute hold). For GC-FID analysis, the flame ionization detector operated at 300°C. GC-FID- and GC-MS-generated peaks were integrated using Hewlett-Packard Chemstation software. Identification of terpenes was based on comparison of retention times and mass spectra with those of authentic standards or with mass spectra in the Wiley library. In order to calculate needle monoterpene concentrations on a ng mg^–1^ dry weight basis, the residue of 100 mg extracted material of each trees was dried and weighed as described by Martin et al., 2002.

### Statistics

The effect of drought treatment was tested using one-way ANOVA. When test results were significant, the means were compared using Tukey’s *post hoc* test at *P* < 0.05. Normality was tested by the Shapiro-Wilk test.

## Results

### Drought Treatment Reduces Photosynthesis and Increases Abscisic Acid (ABA) in White Spruce

**FIGURE 1:**
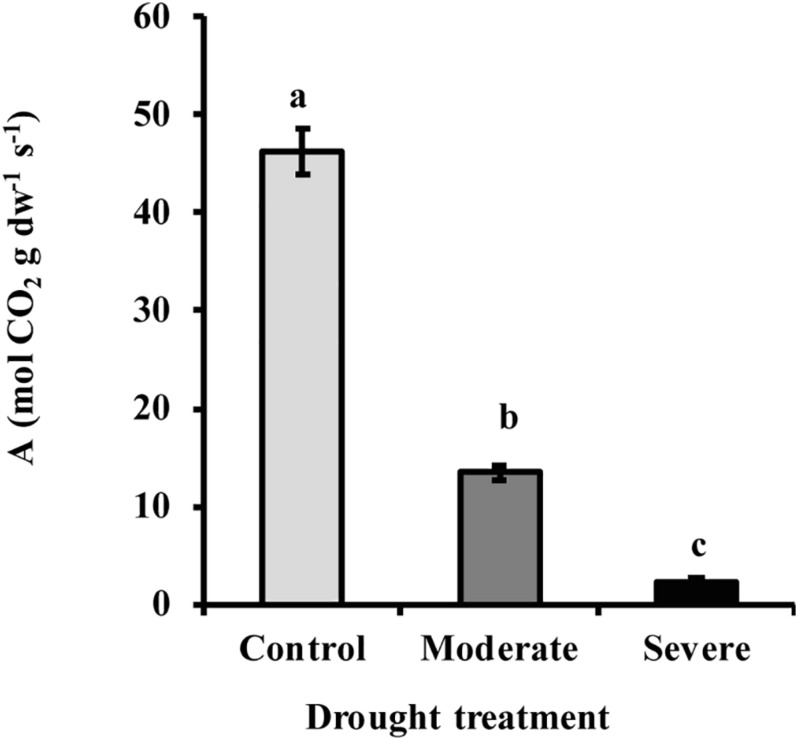
Drought reduced the rate of photosynthesis *A* in white spruce (*Picea glauca*). Photosynthesis declined under moderate (transpiration rate 50% of well-watered control) and severe drought treatment (transpiration rate 20% of control). Data shown are means of five biological replicate ± SE. Different letters indicate significant differences at *P* < 0.05.

**FIGURE 2:**
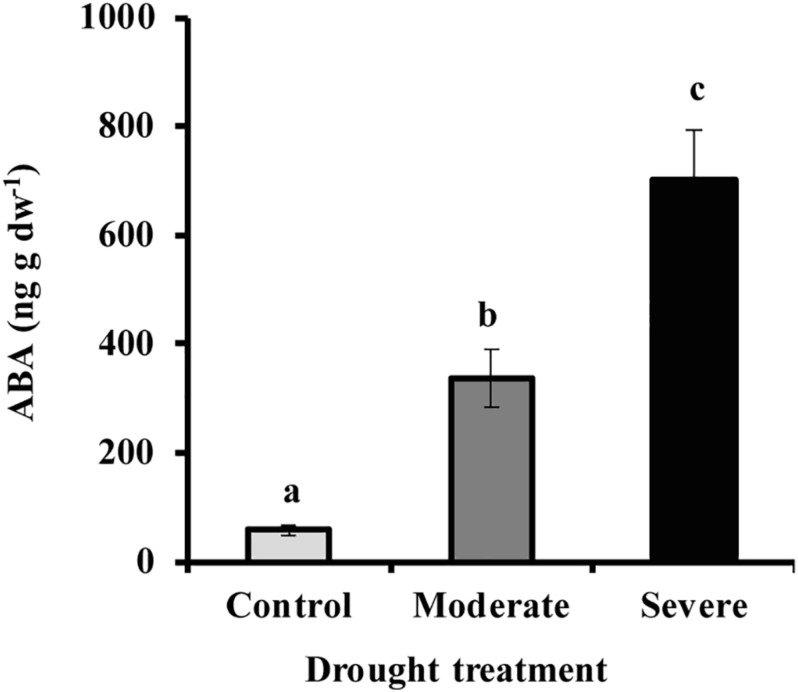
Drought increased needle ABA content under moderate and severe drought conditions. Data shown are means of five biological replicate ± SE. Different letters indicate significant differences at *P* < 0.05.

By withholding water, two different drought treatments were imposed on young *Picea glauca* (white spruce) trees. In the moderate stress treatment, transpiration rate was reduced to 50% of that in well-watered trees, which occurred when the fraction of transpirable soil water (FTSW) was 12%. Under severe stress transpiration rate was reduced to 20% of that in well-watered trees, which occurred when the FTSW was 3% (Supplementary Figure S1). Photosynthesis decreased significantly under drought by an average of 70 and 96% under moderate and severe stress, respectively (Figure 1). At the same time, the hormone abscisic acid (ABA) increased by an average of 6.7- and 12.8-fold, respectively, under moderate and severe stress (Figure 2).

### Drought Treatment Reduces Photosynthetic Incorporation Into Sucrose in Needles, but Not Sucrose Content

To investigate the effect of drought on basic carbohydrate metabolism in white spruce needles, we measured ^13^CO_2_ incorporation into glucose, fructose and sucrose. Incorporation into sucrose declined significantly by an average of 53 and 69% under moderate and severe stress, respectively, as a consequence of reduced photosynthesis, while incorporation into glucose and fructose was not affected (Supplementary Figure S3).

### Drought Reduces Pools of Some MEP Pathway Intermediates and the Emission of the MEP Pathway Product Isoprene

**FIGURE 3:**
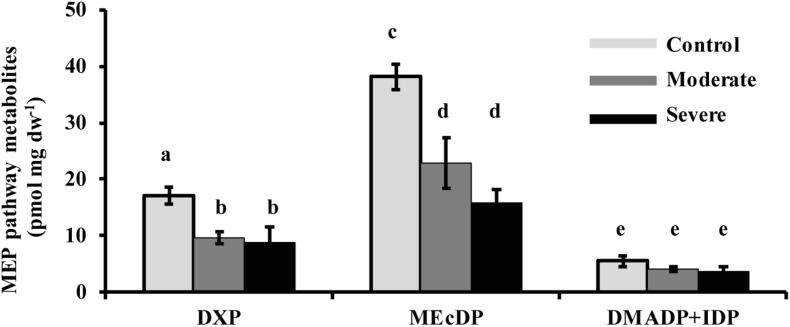
Drought reduced the concentrations of the MEP pathway intermediates, DXP and MEcDP, but not DMADP + IDP in needles. Data shown are means of five biological replicate ± SE. Different letters indicate significant differences at *P* < 0.05.

**FIGURE 4:**
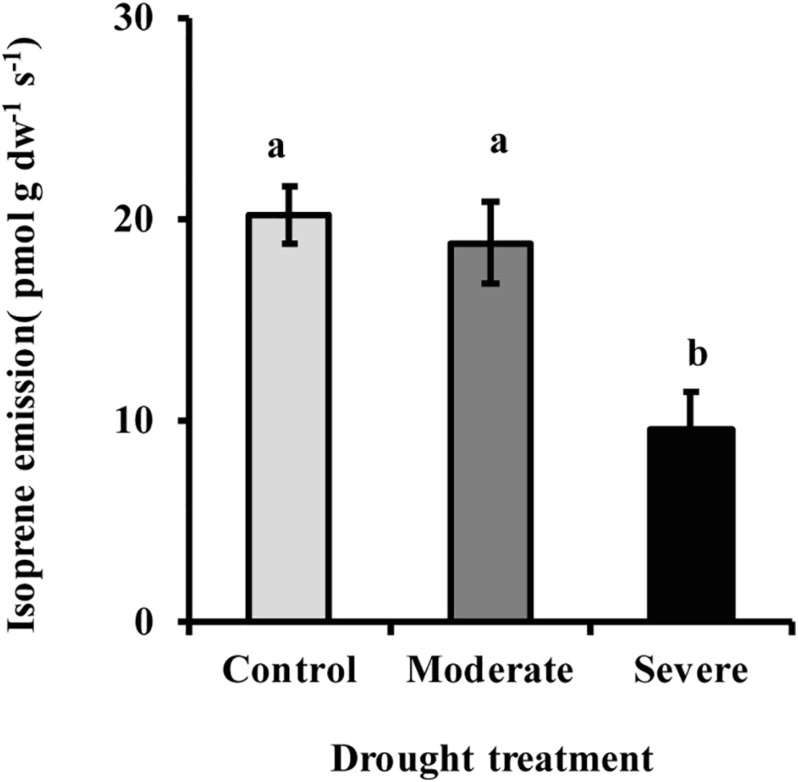
Drought reduced isoprene emission from needles as measured with a PTR-MS. Data shown are means of five biological replicate ± SE. Different letters indicate significant differences at *P* < 0.05.

The MEP pathway intermediates 1-deoxy-D-xylulose 5-phosphate (DXP) and 2-*C*-methyl-D-erythritol-2,4-cyclodiphosphate (MEcDP) declined significantly under moderate and severe drought stress in white spruce (Figure 3). By contrast, the pool of dimethylallyl diphosphate (DMADP) and isopentenyl diphosphate (IDP), present at less than half the level of the other intermediates measured, showed no change under both stress levels. Emission of isoprene, an immediate volatile product of the MEP pathway, was significantly reduced by over half only during severe stress (Figure 4).

### Drought Reduces Relative Incorporation of ^13^CO_2_ Into MEP Pathway Intermediates and Isoprene

**FIGURE 5:**
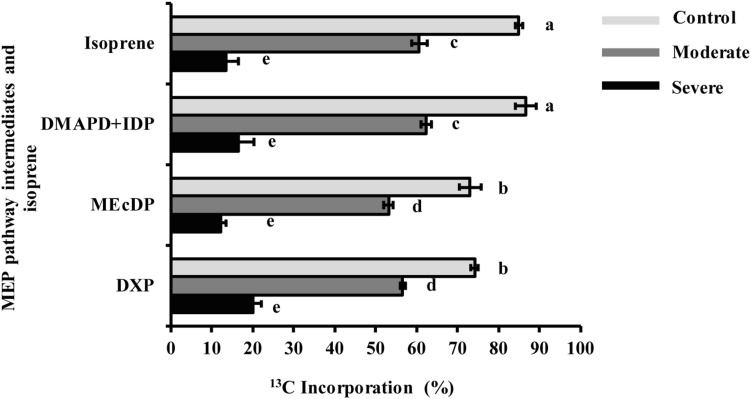
Drought decreased ^13^C incorporation from ^13^CO_2_ into MEP pathway intermediates and isoprene under steady state conditions after 50 min labeling. Data shown are means of five biological replicate ± SE. Different letters indicate significant differences at *P* < 0.05.

During the 50 min time course of ^13^CO_2_ labeling under control conditions, DXP and MEcDP were labeled to nearly 75% while labeling of DMADP + IDP and isoprene reached 87 and 85%, respectively (Figure 5). A typical pattern of isoprene labeling under all condition, is shown in Supplementary Figure S4. Among the isotopologues measured, the fully labeled molecule (*m/z* 74) was the most abundant at all time points after 20 min (Supplementary Figure S5). However, under moderate stress, labeling of DMADP + IDP and isoprene from ^13^CO_2_ was 60%, and DXP and MEcDP still showed significantly lower incorporation than DMADP + IDP and isoprene. Under severe stress, incorporation into all MEP pathway intermediates and isoprene was only 10–20% (Figure 5). Considering isotopologues, the fully labeled molecule was only a minor component under both moderate and severe stress (Supplementary Figure S5). Thus increasing drought led to increased contribution of alternative carbon sources to the MEP pathway, rather than newly made products of photosynthesis.

### Drought Decreases Metabolic Flux Through the MEP Pathway

**FIGURE 6:**
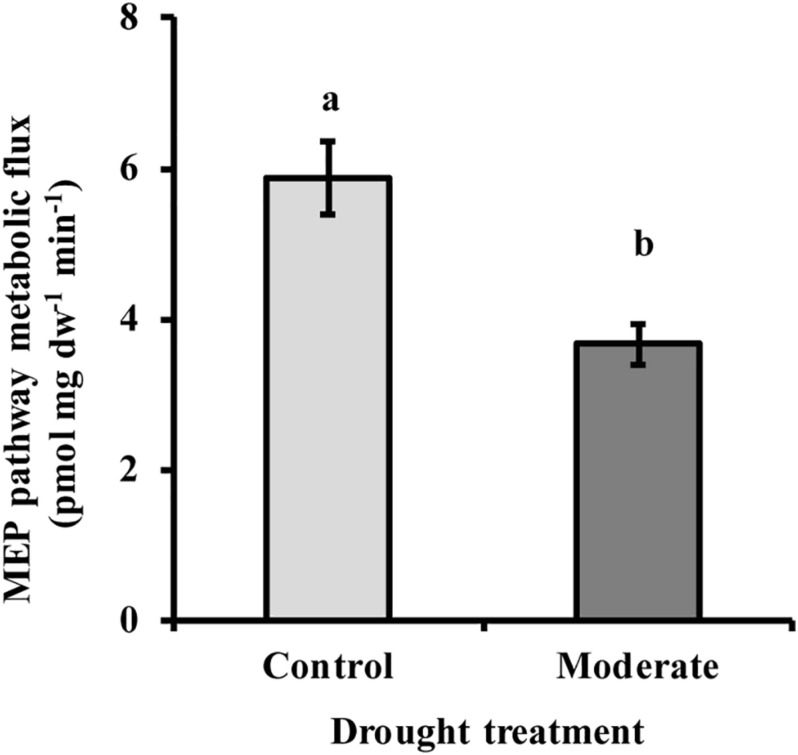
Drought decreased MEP pathway metabolic flux under moderate stress conditions. Under severe stress metabolic flux was not detectable due to very low ^13^C incorporation. Flux was calculated as described in the text. Data shown are means of three biological replicate ± SE. Different letters indicate significant differences at *P* < 0.05.

Metabolic flux through the MEP pathway was calculated from the ^13^C labeling of isoprene from ^13^CO_2_ with time as a function of the pool sizes of the intermediates measured. The labeling of the pathway product isoprene was used since it could be conveniently assessed over the time course and was equivalent to the labeling of the final pathway intermediates, DMADP and IDP (Figure 5), as might be expected since isoprene is produced in a single enzymatic step from DMADP and released from the plant immediately as a volatile gas. Metabolic flux declined significantly (37%) under moderate drought (Figure 6). Under severe drought, metabolic flux could not be calculated because of the lack of sufficient incorporation of ^13^C for accurate measurement.

### Drought Reduces the Activity of 1-deoxy-D-xylulose 5-phosphate Synthase (DXS)

**FIGURE 7:**
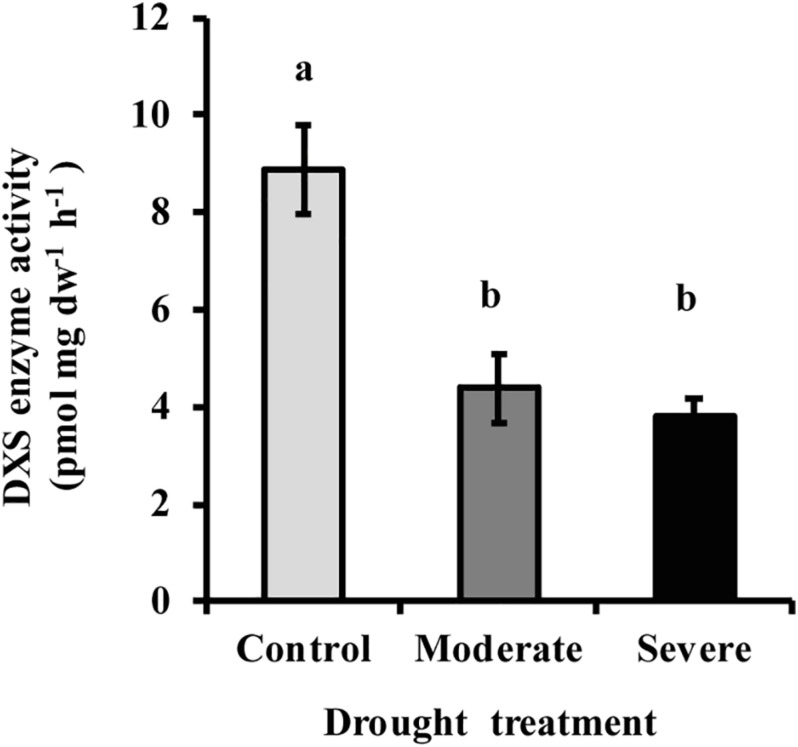
Drought treatment caused a decline in the activity of DXS measured *in vitro* in extracts of white spruce needles. Data shown are means of three biological replicate ± SE. Different letters indicate significant differences at *P* < 0.05.

The first step of the MEP pathway, the condensation of pyruvate and glyceraldehyde 3-phosphate to form DXP, is catalyzed by DXP synthase (DXS). The activity of DXS measured *in vitro* in extracts of freshly ground needles was significantly reduced (50–55%) under both moderate and severe drought treatments, but no significant difference was observed between the two drought levels (Figure 7). This decline was similar to that observed for the product of the enzyme, DXP (Figure 3).

### Drought Affects the Levels of Some Carotenoid Pigments and a Derivative, but Does Not Influence Chlorophyll Content

**FIGURE 8:**
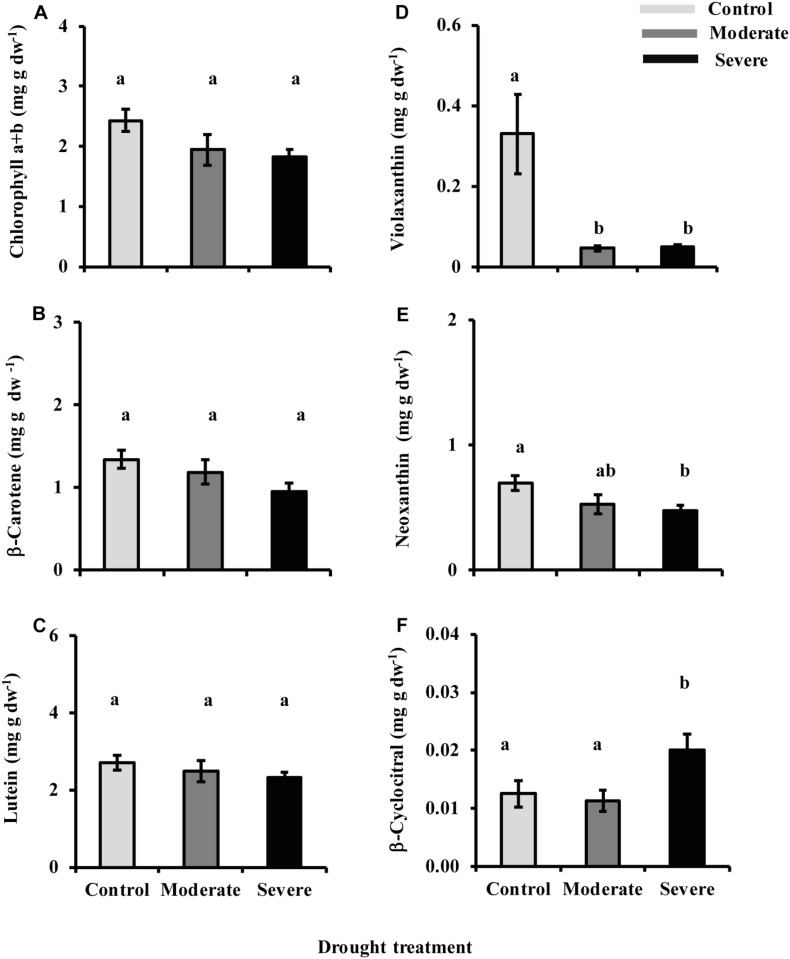
Drought influences chlorophyll and carotenoid levels. Presented are data for **(A)** chlorophyll a and chlorophyll b, **(B)** β-carotene, **(C)** lutein, **(D)** violaxanthin, and **(E)** neoxanthin measured by HPLC-UV, and **(F)** β-cyclocitral measured by LC-MS. Data shown are means of five biological replicate ± SE. Different letters indicate significant differences at *P* < 0.05.

The decrease in metabolic flux through the MEP pathway might be expected to impact the levels of carotenoid and chlorophyll pigments since carotenoids are wholly derived from the MEP pathway while the C_20_ side chain of chlorophylls a and b is synthesized by the MEP pathway. However, the sum of chlorophyll a and chlorophyll b did not show any significant reduction with drought despite a declining trend (ANOVA one-way *P* = 0.109) (Figure 8A), even though photosynthesis itself was strongly reduced under both moderate and severe drought (Figure 1). Among the carotenoids, β-carotene (ANOVA one-way *P* = 0.110) and lutein (ANOVA one way *P* = 0.449) also did not decline significantly, although declining trends were evident (Figures 8B,C). By contrast, the xanthophyll violaxanthin showed a statistically significant reduction under both drought treatments (Figure 8D) while neoxanthin was reduced under severe drought (Figure 8E). An oxidized derivative of β-carotene, β-cyclocitral, that quenches oxidant species during oxidative stress (Ramel et al., 2012) increased under severe drought (Figure 8F).

### Stored Monoterpene Content Is Not Influenced by Drought

Monoterpenes are major constituents of resins of *P. glauca* and other conifers, and are also produced by the MEP pathway. The pool size of stored monoterpenes in needles was not influenced by drought treatments, and the amounts of individual monoterpenes were also unaltered (Supplementary Table S1).

## Discussion

### Decline in MEP Pathway Flux Under Drought Is Partially Mitigated by Use of Carbon Sources Other Than Photosynthesis

Drought treatment of young *Picea glauca* trees in our study caused a range of physiological and metabolic responses. A decline in transpiration rate, 50 and 80% under moderate and severe drought, respectively, coincided with a 72 and 96% decrease in photosynthetic rate, respectively (Figure 1). These changes were likely triggered by increased stomatal closure (Chaves, 1991; Cornic, 2000), induced by a sharp increase in ABA concentration, over 6- and 12-fold, respectively, under moderate and severe stress (Figure 2).

Despite the steep drop in carbon fixation, metabolic flux through the MEP pathway decreased by only 37% at moderate stress. Our ^13^CO_2_ labeling data show that as the photosynthetic rate declined, reliance on alternative carbon sources increased to 40% under moderate stress and to 85–90% under severe stress for both the DMADP + IDP pool and the direct product isoprene (Figure 5 and Supplementary Figure S4). Alternative carbon sources for isoprene have been suggested to include chloroplast starch deposits, CO_2_ recycled by photorespiration and other respiratory processes, and glucose from xylem transport (Kreuzwieser et al., 2002; Loreto et al., 2004; Schnitzler et al., 2004; Jardine et al., 2014).

Based on the identical percentage of ^13^C incorporated in DMADP + IDP and isoprene under all conditions, we assume that the same alternative carbon sources employed for isoprene are also used for the MEP pathway (Figure 5). The use of alternative carbon sources under drought is also suggested by changes in the relative labeling of MEP pathway intermediates from ^13^CO_2_. Both DXP and MEcDP had significantly lower percentages of incorporated ^13^C than DMADP + IDP and isoprene under both control conditions and moderate drought (Figure 5). These data indicate the presence of additional pools of these intermediates outside the chloroplast as previously measured in Arabidopsis for MEcDP (Wright et al., 2014). These additional pools may be located in the cytosol. The export of MEcDP from the plastid to the cytosol has been previously reported (Xiao et al., 2012; Zhou et al., 2012; González-Cabanelas et al., 2015), and this metabolite also participates in retrograde signaling processes from the plastid to the nucleus. Although an extra plastidic pool of DXP was never measured previously, plastidial uptake of exogenous DXP and its non-phosphorylated derivative was shown in *Eucalyptus globulus* and *Arabidopsis thaliana* (Wolfertz et al., 2003, 2004; Hemmerlin et al., 2006), and a plastidial transporter capable of accepting DXP was described in spinach (Flügge and Gao, 2005). Under severe drought DXP and MEcDP had similar ^13^C labeling percentages as DMADP + IDP and isoprene (Figure 5), pointing to the disappearance of these additional pools and suggesting that the intermediates are now localized exclusively in the chloroplast. These additional quantities of MEP intermediates may help buffer the pathway against declines in metabolic flux under drought conditions caused by the decrease in photosynthetic rate. Further investigation on the intracellular trafficking of DXP and MEcDP may shed light on new mechanisms controlling the MEP pathway during stress.

The mevalonate (MVA) pathway of isoprenoid biosynthesis, localized in the cytosol, ER and peroxisomes, also produces DMADP and IDP. However, our data give no support to a role for products of the MVA pathway in supplying the MEP pathway under stress. Import of DMADP or IDP from outside the plastid would decrease ^13^C incorporation in these diphosphate intermediates and in the DMADP product isoprene. Yet under drought there was no significant decline in ^13^C labeling of either the DMADP + IDP pool or isoprene relative to the earlier MEP pathway intermediates, and sometimes even an increase (Figure 5). Thus the plastids did not import a supply of prenyl diphosphate intermediates under drought. Moreover, given that DMADP + IDP and isoprene always had the same ^13^C incorporation percentage under all treatments, one can conclude that the only detectable pool of DMADP in the cell must serve as a precursor to isoprene synthesis, and therefore belongs to the MEP pathway and resides in the plastid where isoprene is made. This suggests that the MVA pathway is not operating at all under our experimental conditions, (see section “Materials and Methods” for details). Similar ^13^CO_2_ measurements of the illuminated rosettes of *Arabidopsis thaliana* conducted by Wright et al., 2014 also found no evidence for measurable pools of DMADP outside the plastid that could be attributed to the MVA pathway. This previous study also found no evidence for labeling of MVA pathway intermediates from ^13^CO_2_ in illuminated *A. thaliana* rosettes. The MVA pathway is also known from other studies to be less active during the day due to the negative effect of light on the transcription of pathway genes (Learned and Connolly, 1997; Rodríguez-Concepción, 2006; Vranová et al., 2013).

### Down-Regulation of MEP Pathway Flux During Moderate Drought May Be Mediated by the Enzyme DXS

The down-regulation of the MEP pathway we observed under moderate drought was exhibited not only in the 37% decline of the metabolic flux (Figure 6), but also by declines of 40–50% in the levels of the key intermediates, DXP and MEcDP (Figure 3). In considering the mechanism for MEP pathway reduction, we focused on DXS, the first enzyme of the sequence. DXS was shown to be the principal rate-controlling step of the pathway in photosynthetic tissue of *Arabidopsis thaliana* based on metabolic control analysis (Wright et al., 2014). In addition, increases in *DXS* gene transcript levels were found to correlate with higher accumulation of MEP pathway end products (Estévez et al., 2001). Here we demonstrated that DXS activity was reduced by about 50–55% under moderate stress (Figure 7), very similar to the decline in metabolic flux, suggesting that this enzyme may well have modulated the down-regulation of the pathway seen under these conditions. Regulation of DXS activity also occurs post-transcriptionally (Rodríguez-Concepción, 2006; Hemmerlin, 2013; Banerjee and Sharkey, 2014). For example, DXS protein levels can be modulated by the casein lytic proteinase (CLP) complex (Pulido et al., 2016), which responds to changing environmental conditions. In particular, long term stress and drought, have been shown to increase some component of the CLP protein complex (Zheng et al., 2002; Demirevska et al., 2008), which could increase the removal of DXS during the process of protein quality control (Flores-Pérez et al., 2008). In addition, DXS activity can also be controlled by feedback inhibition of the MEP pathway by the end product DMADP (Banerjee et al., 2013; Ghirardo et al., 2014). Further work is needed to clarify the mechanism by which DXS catalysis is altered under drought conditions.

1-deoxy-D-xylulose-5-phosphate synthase does not appear to have a large role under severe drought since its activity did not significantly change between moderate and severe drought conditions (Figure 7), while the metabolic flux was reduced to a level that was not measurable. This result suggests that the MEP pathway is regulated in a very different manner under severe drought than under moderate drought. An enzyme one step beyond the MEP pathway that could also have a regulatory impact is isoprene synthase (Brilli et al., 2007). Inhibition of this reaction would reduce isoprene formation, as happened under severe drought in this study (Figure 4), and result in the allocation of DMADP to other MEP pathway products. Other pathway enzymes may also exert more control, such as 1-deoxy-D-xylulose 5-phosphate reductoisomerase (DXR) (Carretero-Paulet et al., 2006), 4-hydroxy-3-methylbut-2-en-1-yl diphosphate synthase (HDS) (Wang et al., 2019) and 4-hydroxy-3-methylbut-2-enyl diphosphate reductase, HDR.

### Drought Effects on the Levels of MEP Pathway Products

Despite the substantial decline in MEP pathway flux under drought, we found no commensurate reduction in the major products of the MEP pathway produced in photosynthetic cells, the chlorophylls, lutein and β-carotene. Depending on the turnover rate of these pigments, it is possible that the drought treatment was not long enough to observe any net depletion. Some depletion of β-carotene is indicated by the sharp increase in β-cyclocitral, a β-carotene oxidation product (Figure 8F). However, the β-cyclocitral detected represents just a few percent of the total β-carotene pool. The decline in the carotenoid violaxanthin was considerable under both stress treatments but this is associated with the activation of the xanthophyll cycle to dissipate excess radiant energy via non-photochemical quenching and prevent the formation of reactive oxygen species (Demmig-Adams and Adams III, 1996). Other MEP pathway-derived products that we did not measure may have been reduced under drought, such as the tocopherols and the prenylquinones: plastoquinones, phylloquinones, and ubiquinones.

Under severe drought (but not under moderate drought), there was a very significant reduction (>50%) in the MEP pathway product isoprene. This may have diverted enough MEP pathway flux to chlorophyll and carotenoid formation to keep the pools of these pigments stable. However, transgenic silencing of isoprene formation in poplar led to only slight increases in the levels of chlorophylls and carotenoids (Behnke et al., 2007; Ghirardo et al., 2014), but the outcome could be different under severe drought. Under neither severe drought nor moderate drought was there any significant reduction in stored monoterpene formation. These findings are in agreement with previous reports about the general lack of monoterpene metabolism in conifer needles late in the growing season. For example, in *Picea abies*, the size of the stored pool of monoterpenes in current year needles did not change after the first 2 months of growth in July, and did not change in older needles at all over the entire growing season (Schönwitz et al., 1990). Our experiment was conducted in August. Exposing *Picea abies* to a low atmospheric CO_2_ concentration (50 ppm) also did not change the amount of monoterpenes stored in current year needles (Huang et al., 2018).

### Isoprene and Other MEP Pathway Products May Help Alleviate the Effects of Drought

The physiology and function of isoprene have been studied for many years since isoprene, produced especially in woody plants (Loreto and Fineschi, 2015), is the most abundant hydrocarbon released into the atmosphere from the earth’s vegetation (Sharkey and Yeh, 2001). Knowledge of isoprene physiology and response to environment in conifers is not as large as in angiosperms. Here we monitored isoprene biosynthesis and emission in detail in white spruce under moderate and severe drought. After ^13^CO_2_ incorporation, the percentage of isotopic label in isoprene was virtually identical to the percentage of labeling in the DMADP + IDP pool under all conditions (Figure 5), confirming that DMADP is the biosynthetic source of isoprene. Furthermore, isoprene is formed directly from DMADP by a single reaction catalyzed by isoprene synthase and represents an efficient probe for the operation of the MEP pathway. The percentage of ^13^C labeling in isoprene from ^13^CO_2_ reached 85% after a 50 min time course under steady state conditions. The major role for photosynthesis in providing fixed carbon for isoprene biosynthesis, previously demonstrated in angiosperms (Brilli et al., 2007), is here demonstrated for a gymnosperm, *P. glauca*.

The continued production of isoprene under moderate drought suggests that its function is still needed despite the decline in photosynthetic carbon availability that occurs under these conditions. Isoprene has long been suggested to protect plants against high temperature and oxidative stress by various mechanisms (Singsaas et al., 1997; Vickers et al., 2009; Velikova et al., 2011; Pollastri et al., 2014), most recently by preserving thylakoid membrane stability and elasticity (Pollastri et al., 2019). However, a recent publication has suggested that isoprene may not be abundant enough to function in these ways and may instead act as a general signal for increased abiotic stress tolerance (Zuo et al., 2019). Regardless of function, isoprene formation and emission declined steeply under severe drought in our experiment, a pattern seen in angiosperms as well (Funk et al., 2004; Pegoraro et al., 2004; Brilli et al., 2007). Since continued emission under moderate stress, but abrupt decline at higher stress is widespread for isoprene emission in plants, further study of the causes behind this pattern may help to shed more light on isoprene function and MEP pathway regulation.

Changes in the levels of other isoprenoids under drought in this study may also help to alleviate stresses associated with low water supply. For example, the decline in the carotenoid violaxanthin is associated with the activation of the xanthophyll cycle. The increase in β-cyclocitral under severe drought may also help enhance tolerance toward oxidative stress after its conversion to β-cyclocitric acid (D’Alessandro et al., 2019). Although we did not measure tocopherols, these isoprenoid antioxidants could also reduce oxidative stress. Evidence for the alleviation of oxidative stress during drought comes from the lack of accumulation of MEcDP under these conditions. Previous studies showed an accumulation of MEcDP during high light as a consequence of the susceptibility to oxidative stress of the [4Fe–4S]-cluster contained in the following enzyme 4-hydroxy-3-methylbut-2-en-1-yl diphosphate synthase (HDS) (Rivasseau et al., 2009). By contrast, in our experiment the MEcDP pool size was not increased at all, but reduced. It is clear that the continued operation of the MEP pathway during drought may make a critical contribution to plant survival.

## Conclusion

Under drought, white spruce trees significantly decrease their metabolic flux through the MEP pathway, but this decrease is not nearly as pronounced as the decrease in photosynthetic carbon fixation and transpiration rate. Reliance on alternative carbon sources besides photosynthesis is considerable under drought, and contributes to the continued operation of the MEP pathway. However, the other isoprenoid pathway (the MVA pathway) was not one of these alternative sources. More investigations are needed to determine how alternative carbon sources are recruited to the MEP pathway under stress and how this is regulated. The relative importance of the MEP pathway under drought may be a consequence of the number of pathway products shown to help protect against drought-associated oxidative stresses, including carotenoids, tocopherols and isoprene (Mattos and Moretti, 2015; Zuo et al., 2019). Control of MEP flux under moderate drought may be maintained by the well-known pathway regulator DXS. Under severe drought, when the DXS enzyme exerts a reduced role and isoprene emission drops, regulatory mechanisms could involve other MEP pathway enzymes. Further research is necessary to determine how a pathway that produces so many anti-oxidant metabolites is kept in service under drought. As the world’s climate warms, such knowledge may be especially valuable for boreal tree species, such as the white spruce.

## Data Availability Statement

All datasets presented in this study are included in the article/Supplementary Material.

## Author Contributions

EP and LW designed the experiments. EP performed the experiments, analyzed the data, and wrote the manuscript. JR performed the calculation of the metabolic flux. JR, DG-C, FL, AS, JG, and LW supervised the study and complemented writing. All authors contributed to the article and approved the submitted version.

## Conflict of Interest

The authors declare that the research was conducted in the absence of any commercial or financial relationships that could be construed as a potential conflict of interest.

## References

[B1] Ahkami A. H. Wang W. Wietsma T. W. Winkler T. Lange I. Jansson C. (2019). Metabolic shifts associated with drought-induced senescence in *Brachypodium*. *Plant Sci.* 289:110278. 10.1016/j.plantsci.2019.110278 31623774

[B2] Banerjee A. Sharkey T. (2014). Methylerythritol 4-phosphate (MEP) pathway metabolic regulation. *Nat. Prod. Rep* 31 1043–1055. 10.1039/c3np70124g 24921065

[B3] Banerjee A. Wu Y. Banerjee R. Li Y. Yan H. Sharkey T. D. (2013). Feedback inhibition of deoxy-D-xylulose-5-phosphate synthase regulates the methylerythritol 4-phosphate pathway. *J. Biol. Chem.* 288 16926–16936. 10.1074/jbc.m113.464636 23612965 PMC3675625

[B4] Behnke K. Ehlting B. Teuber M. Bauerfeind M. Louis S. Hänsch R. (2007). Transgenic, non-isoprene emitting poplars don’t like it hot. *Plant J.* 51 485–499. 10.1111/j.1365-313x.2007.03157.x 17587235

[B5] Brilli F. Barta C. Fortunati A. Lerdau M. Loreto F. Centritto M. (2007). Response of isoprene emission and carbon metabolism to drought in white poplar (*Populus alba*) saplings. *New Phytol.* 175 244–254. 10.1111/j.1469-8137.2007.02094.x 17587373

[B6] Carretero-Paulet L. Cairo A. Botella-Pavía P. Besumbes O. Campos N. Boronat A. (2006). Enhanced flux through the methylerythritol 4-phosphate pathway in Arabidopsis plants overexpressing deoxyxylulose 5-phosphate reductoisomerase. *Plant Mol. Biol.* 62 683–695. 10.1007/s11103-006-9051-9 16941216

[B7] Chaves M. (1991). Effects of water deficits on carbon assimilation. *J. Exp. Bot.* 42 1–16. 10.1093/jxb/42.1.1

[B8] Cornic G. (2000). Drought stress inhibits photosynthesis by decreasing stomatal aperture–not by affecting ATP synthesis. *Trends Plant Sci.* 5 187–188. 10.1016/s1360-1385(00)01625-3

[B9] D’Alessandro S. Mizokami Y. Legeret B. Havaux M. (2019). The apocarotenoid β-cyclocitric acid elicits drought tolerance in plants. *Iscience* 19 461–473. 10.1016/j.isci.2019.08.003 31437750 PMC6710299

[B10] Demirevska K. Simova-Stoilova L. Vassileva V. Feller U. (2008). Rubisco and some chaperone protein responses to water stress and rewatering at early seedling growth of drought sensitive and tolerant wheat varieties. *Plant Growth Regul.* 56 97–106. 10.1007/s10725-008-9288-1

[B11] Demmig-Adams B. Adams W. W. III (1996). The role of xanthophyll cycle carotenoids in the protection of photosynthesis. *Trends Plant Sci.* 1 21–26. 10.1016/S1360-1385(96)80019-7

[B12] Dudareva N. Andersson S. Orlova I. Gatto N. Reichelt M. Rhodes D. (2005). The nonmevalonate pathway supports both monoterpene and sesquiterpene formation in snapdragon flowers. *Proc. Natl. Acad. Sci. U.S.A.* 102 933–938. 10.1073/pnas.0407360102 15630092 PMC545543

[B13] Eberl F. Perreca E. Vogel H. Wright L. P. Hammerbacher A. Veit D. (2018). Rust infection of black poplar trees reduces photosynthesis but does not affect isoprene biosynthesis or emission. *Front. Plant Sci.* 9:1733. 10.3389/fpls.2018.01733 30538714 PMC6277707

[B14] Enfissi E. M. Fraser P. D. Lois L. M. Boronat A. Schuch W. Bramley P. M. (2005). Metabolic engineering of the mevalonate and non-mevalonate isopentenyl diphosphate-forming pathways for the production of health-promoting isoprenoids in tomato. *Plant Biotechnol. J.* 3 17–27. 10.1111/j.1467-7652.2004.00091.x 17168896

[B15] Estévez J. M. Cantero A. Reindl A. Reichler S. León P. (2001). 1-Deoxy-D-xylulose-5-phosphate synthase, a limiting enzyme for plastidic isoprenoid biosynthesis in plants. *J. Biol. Chem.* 276 22901–22909. 10.1074/jbc.m100854200 11264287

[B16] Flores-Pérez Ú Sauret-Güeto S. Gas E. Jarvis P. Rodríguez-Concepción M. (2008). A mutant impaired in the production of plastome-encoded proteins uncovers a mechanism for the homeostasis of isoprenoid biosynthetic enzymes in Arabidopsis plastids. *Plant Cell* 20 1303–1315. 10.1105/tpc.108.058768 18469163 PMC2438453

[B17] Flügge U.-I. Gao W. (2005). Transport of isoprenoid intermediates across chloroplast envelope membranes. *Plant Biol.* 7 91–97. 10.1055/s-2004-830446 15666208

[B18] Funk J. Mak J. Lerdau M. (2004). Stress-induced changes in carbon sources for isoprene production in *Populus deltoides*. *Plant Cell Environ.* 27 747–755. 10.1111/j.1365-3040.2004.01177.x

[B19] Ghirardo A. Wright L. P. Bi Z. Rosenkranz M. Pulido P. Rodríguez-Concepción M. (2014). Metabolic flux analysis of plastidic isoprenoid biosynthesis in poplar leaves emitting and nonemitting isoprene. *Plant Physiol.* 165 37–51. 10.1104/pp.114.236018 24590857 PMC4012595

[B20] González-Cabanelas D. Wright L. P. Paetz C. Onkokesung N. Gershenzon J. Rodríguez-Concepción M. (2015). The diversion of 2-C-methyl-d-erythritol-2, 4-cyclodiphosphate from the 2-C-methyl-d-erythritol 4-phosphate pathway to hemiterpene glycosides mediates stress responses in *Arabidopsis thaliana*. *Plant J.* 82 122–137. 10.1111/tpj.12798 25704332

[B21] Guo R. Shi L. Jiao Y. Li M. Zhong X. Gu F. (2018). Metabolic responses to drought stress in the tissues of drought-tolerant and drought-sensitive wheat genotype seedlings. *AoB Plants* 10:ly016. 10.1093/aobpla/ply016 PMC5881611 29623182

[B22] Hemmerlin A. (2013). Post-translational events and modifications regulating plant enzymes involved in isoprenoid precursor biosynthesis. *Plant Sci.* 203 41–54. 10.1016/j.plantsci.2012.12.008 23415327

[B23] Hemmerlin A. Harwood J. L. Bach T. J. (2012). A raison d’être for two distinct pathways in the early steps of plant isoprenoid biosynthesis? *Prog. Lipid Res.* 51 95–148. 10.1016/j.plipres.2011.12.001 22197147

[B24] Hemmerlin A. Tritsch D. Hartmann M. Pacaud K. Hoeffler J.-F. van Dorsselaer A. (2006). A cytosolic Arabidopsis D-xylulose kinase catalyzes the phosphorylation of 1-deoxy-D-xylulose into a precursor of the plastidial isoprenoid pathway. *Plant Physiol.* 142 441–457. 10.1104/pp.106.086652 16920870 PMC1586049

[B25] Huang J. Hartmann H. Hellén H. Wisthaler A. Perreca E. Weinhold A. (2018). New perspectives on CO2, temperature, and light effects on BVOC emissions using online measurements by PTR-MS and cavity ring-down spectroscopy. *Environ. Sci. Technol.* 52 13811–13823. 30335995 10.1021/acs.est.8b01435

[B26] Jardine K. Chambers J. Alves E. G. Teixeira A. Garcia S. Holm J. (2014). Dynamic balancing of isoprene carbon sources reflects photosynthetic and photorespiratory responses to temperature stress. *Plant Physiol.* 166 2051–2064. 10.1104/pp.114.247494 25318937 PMC4256868

[B27] Kreuzwieser J. Graus M. Wisthaler A. Hansel A. Rennenberg H. Schnitzler J. P. (2002). Xylem-transported glucose as an additional carbon source for leaf isoprene formation in Quercus robur. *New Phytol.* 156 171–178. 10.1046/j.1469-8137.2002.00516.x 33873274

[B28] Laule O. Fürholz A. Chang H.-S. Zhu T. Wang X. Heifetz P. B. (2003). Crosstalk between cytosolic and plastidial pathways of isoprenoid biosynthesis in *Arabidopsis thaliana*. *Proc. Natl. Acad. Sci. U.S.A.* 100 6866–6871. 10.1073/pnas.1031755100 12748386 PMC164538

[B29] Learned R. M. Connolly E. L. (1997). Light modulates the spatial patterns of 3-hydroxy-3-methylglutaryl coenzyme A reductase gene expression in *Arabidopsis thaliana*. *Plant J.* 11 499–511. 10.1046/j.1365-313x.1997.11030499.x 9107038

[B30] Lindinger W. Hansel A. Jordan A. (1998). On-line monitoring of volatile organic compounds at pptv levels by means of proton-transfer-reaction mass spectrometry (PTR-MS) medical applications, food control and environmental research. *Int. J. Mass Spectrom. Ion Process.* 173 191–241. 10.1016/s0168-1176(97)00281-4

[B31] Loreto F. Fineschi S. (2015). Reconciling functions and evolution of isoprene emission in higher plants. *New Phytol.* 206 578–582. 10.1111/nph.13242 25557381

[B32] Loreto F. Pinelli P. Brancaleoni E. Ciccioli P. (2004). 13C labeling reveals chloroplastic and extrachloroplastic pools of dimethylallyl pyrophosphate and their contribution to isoprene formation. *Plant Physiol.* 135 1903–1907. 10.1104/pp.104.039537 15286296 PMC520762

[B33] Martin D. Tholl D. Gershenzon J. Bohlmann J. (2002). Methyl jasmonate induces traumatic resin ducts, terpenoid resin biosynthesis, and terpenoid accumulation in developing xylem of Norway spruce stems. *Plant Physiol.* 129 1003–1018. 10.1104/pp.011001 12114556 PMC166496

[B34] Mattos L. Moretti C. (2015). Oxidative stress in plants under drought conditions and the role of different enzymes. *Enzym. Eng.* 5:136.

[B35] Mundim F. M. Pringle E. G. (2018). Whole-plant metabolic allocation under water stress. *Front. Plant Sci.* 9:852. 10.3389/fpls.2018.00852 29988542 PMC6026660

[B36] Newville M. Stensitzki T. Allen D. Ingargiola A. (2014). *LMFIT: Non-Linear Least-Square Minimization and Curve-Fitting for Python (Version 0.8.0).* 10.5281/zenodo.11813

[B37] Onkokesung N. Reichelt M. Wright L. P. Phillips M. A. Gershenzon J. Dicke M. (2019). The plastidial metabolite 2-C-methyl-D-erythritol-2, 4-cyclodiphosphate modulates defence responses against aphids. *Plant Cell Environ.* 42 2309–2323. 10.1111/pce.13538 30786032 PMC6850158

[B38] Pegoraro E. Rey A. Greenberg J. Harley P. Grace J. Malhi Y. (2004). Effect of drought on isoprene emission rates from leaves of *Quercus virginiana* Mill. *Atmos. Environ.* 38 6149–6156. 10.1016/j.atmosenv.2004.07.028

[B39] Pollastri S. Jorba I. Hawkins T. J. Llusià J. Michelozzi M. Navajas D. (2019). Leaves of isoprene-emitting tobacco plants maintain PSII stability at high temperatures. *New Phytol.* 223 1307–1318. 10.1111/nph.15847 30980545

[B40] Pollastri S. Tsonev T. Loreto F. (2014). Isoprene improves photochemical efficiency and enhances heat dissipation in plants at physiological temperatures. *J. Exp. Bot.* 65 1565–1570. 10.1093/jxb/eru033 24676032 PMC3967094

[B41] Price D. T. Alfaro R. Brown K. Flannigan M. Fleming R. Hogg E. (2013). Anticipating the consequences of climate change for Canada’s boreal forest ecosystems. *Environ. Rev.* 21 322–365. 10.1139/er-2013-0042

[B42] Pulido P. Llamas E. Llorente B. Ventura S. Wright L. P. Rodríguez-Concepción M. (2016). Specific Hsp100 chaperones determine the fate of the first enzyme of the plastidial isoprenoid pathway for either refolding or degradation by the stromal Clp protease in Arabidopsis. *PLoS Genet.* 12:e1005824. 10.1371/journal.pgen.1005824 26815787 PMC4729485

[B43] Ramel F. Birtic S. Cuiné S. Triantaphylidès C. Ravanat J.-L. Havaux M. (2012). Chemical quenching of singlet oxygen by carotenoids in plants. *Plant Physiol.* 158 1267–1278. 10.1104/pp.111.182394 22234998 PMC3291260

[B44] Ray J. D. Sinclair T. R. (1998). The effect of pot size on growth and transpiration of maize and soybean during water deficit stress. *J. Exp. Bot.* 49 1381–1386. 10.1093/jxb/49.325.1381 12432039

[B45] Rivasseau C. Seemann M. Boisson A. M. Streb P. Gout E. Douce R. (2009). Accumulation of 2-C-methyl-D-erythritol 2, 4-cyclodiphosphate in illuminated plant leaves at supraoptimal temperatures reveals a bottleneck of the prokaryotic methylerythritol 4-phosphate pathway of isoprenoid biosynthesis. *Plant Cell Environ.* 32 82–92. 10.1111/j.1365-3040.2008.01903.x 19021881

[B46] Rodríguez-Concepción M. (2006). Early steps in isoprenoid biosynthesis: multilevel regulation of the supply of common precursors in plant cells. *Phytochem. Rev.* 5 1–15. 10.1007/s11101-005-3130-4

[B47] Rodríguez-Concepción M. Forés O. Martínez-García J. F. González V. Phillips M. A. Ferrer A. (2004). Distinct light-mediated pathways regulate the biosynthesis and exchange of isoprenoid precursors during Arabidopsis seedling development. *Plant Cell* 16 144–156. 10.1105/tpc.016204 14660801 PMC301401

[B48] Schnitzler J.-P. Graus M. Kreuzwieser J. Heizmann U. Rennenberg H. Wisthaler A. (2004). Contribution of different carbon sources to isoprene biosynthesis in poplar leaves. *Plant Physiol.* 135 152–160. 10.1104/pp.103.037374 15122010 PMC429343

[B49] Schönwitz R. Lohwasser K. Kloos M. Ziegler H. (1990). Seasonal variation in the monoterpenes in needles of *Picea abies* (L.) *Karst*. *Trees* 4 34–40.

[B50] Selmar D. (2008). Potential of salt and drought stress to increase pharmaceutically significant secondary compounds in plants. *Landbauforschung Volkenrode* 58 139–144.

[B51] Sharkey T. D. Yeh S. (2001). Isoprene emission from plants. *Annu. Rev. Plant Biol.* 52 407–436. 10.1146/annurev.arplant.52.1.407 11337404

[B52] Sinclair T. Ludlow M. (1986). Influence of soil water supply on the plant water balance of four tropical grain legumes. *Funct. Plant Biol.* 13 329–341. 10.1071/pp9860329

[B53] Singsaas E. L. Lerdau M. Winter K. Sharkey T. D. (1997). Isoprene increases thermotolerance of isoprene-emitting species. *Plant Physiol.* 115 1413–1420. 10.1104/pp.115.4.1413 12223874 PMC158606

[B54] Soja A. J. Tchebakova N. M. French N. H. Flannigan M. D. Shugart H. H. Stocks B. J. (2007). Climate-induced boreal forest change: predictions versus current observations. *Glob. Planet. Change* 56 274–296. 10.1016/j.gloplacha.2006.07.028

[B55] Velikova V. Várkonyi Z. Szabó M. Maslenkova L. Nogues I. Kovács L. (2011). Increased thermostability of thylakoid membranes in isoprene-emitting leaves probed with three biophysical techniques. *Plant Physiol.* 157 905–916. 10.1104/pp.111.182519 21807886 PMC3192565

[B56] Vickers C. E. Gershenzon J. Lerdau M. T. Loreto F. (2009). A unified mechanism of action for volatile isoprenoids in plant abiotic stress. *Nat. Chem. Biol.* 5 283–291. 10.1038/nchembio.158 19377454

[B57] Vranová E. Coman D. Gruissem W. (2013). Network analysis of the MVA and MEP pathways for isoprenoid synthesis. *Annu. Rev. Plant Biol.* 64 665–700. 10.1146/annurev-arplant-050312-120116 23451776

[B58] Wang J.-Z. Lei Y. Xiao Y. He X. Liang J. Jiang J. (2019). Uncovering the functional residues of Arabidopsis isoprenoid biosynthesis enzyme HDS. *Proc. Natl. Acad. Sci. U.S.A.* 117 355–361. 10.1073/pnas.1916434117 31879352 PMC6955319

[B59] Wolfertz M. Sharkey T. Boland W. Kühnemann F. Yeh S. Weise S. (2003). Biochemical regulation of isoprene emission. *Plant Cell Environ.* 26 1357–1364. 10.1046/j.0016-8025.2003.01059.x

[B60] Wolfertz M. Sharkey T. D. Boland W. Kühnemann F. (2004). Rapid regulation of the methylerythritol 4-phosphate pathway during isoprene synthesis. *Plant Physiol.* 135 1939–1945. 10.1104/pp.104.043737 15286290 PMC520765

[B61] Wright L. P. Phillips M. A. (2014). Measuring the activity of 1-deoxy-D-xylulose 5-phosphate synthase, the first enzyme in the MEP pathway, in plant extracts. *Methods Mol. Biol.* 1153 9–20. 10.1007/978-1-4939-0606-2_2 24777787

[B62] Wright L. P. Rohwer J. M. Ghirardo A. Hammerbacher A. Ortiz-Alcaide M. Raguschke B. (2014). Deoxyxylulose 5-phosphate synthase controls flux through the methylerythritol 4-phosphate pathway in Arabidopsis. *Plant Physiol.* 165 1488–1504. 10.1104/pp.114.245191 24987018 PMC4119033

[B63] Xiao Y. Savchenko T. Baidoo E. E. Chehab W. E. Hayden D. M. Tolstikov V. (2012). Retrograde signaling by the plastidial metabolite MEcPP regulates expression of nuclear stress-response genes. *Cell* 149 1525–1535. 10.1016/j.cell.2012.04.038 22726439

[B64] Yuan J. Bennett B. D. Rabinowitz J. D. (2008). Kinetic flux profiling for quantitation of cellular metabolic fluxes. *Nat. Protoc.* 3 1328–1340. 10.1038/nprot.2008.131 18714301 PMC2710581

[B65] Zheng B. Halperin T. Hruskova-Heidingsfeldova O. Adam Z. Clarke A. K. (2002). Characterization of chloroplast Clp proteins in Arabidopsis: localization, tissue specificity and stress responses. *Physiol. Plant.* 114 92–101. 10.1034/j.1399-3054.2002.1140113.x 11982939

[B66] Zhou K. Zou R. Stephanopoulos G. Too H.-P. (2012). Metabolite profiling identified methylerythritol cyclodiphosphate efflux as a limiting step in microbial isoprenoid production. *PLoS One* 7:e47513. 10.1371/journal.pone.0047513 23133596 PMC3487848

[B67] Zuo Z. Weraduwage S. M. Lantz A. T. Sanchez L. M. Weise S. E. Wang J. (2019). Isoprene acts as a signaling molecule in gene networks important for stress responses and plant growth. *Plant Physiol.* 180 124–152. 10.1104/pp.18.01391 30760638 PMC6501071

