# Supplementary material for: Effect of Drought on the Methylerythritol 4-Phosphate (MEP) Pathway in the Isoprene Emitting Conifer Picea glauca
Source: Front Plant Sci. 2020 Oct 9;11:546295. doi: 10.3389/fpls.2020.546295 (PMC7581940; doi:10.3389/fpls.2020.546295)
Supplement: Supplementary file 1 [file Data_Sheet_1.PDF]

Supplemental Material

Effect of drought on the methylerythritol 4-phosphate (MEP) pathway in the isoprene emitting conifer *Picea glauca*.

Erica Perreca[1*], Johann Rohwer[2], Diego González-Cabanelas[1], Francesco Loreto[3], Axel Schmidt[1], Jonathan Gershenzon[1], Louwrance Peter Wright[1,4]

*Correspondence

Erica Perreca

eperreca@ice.mpg.de

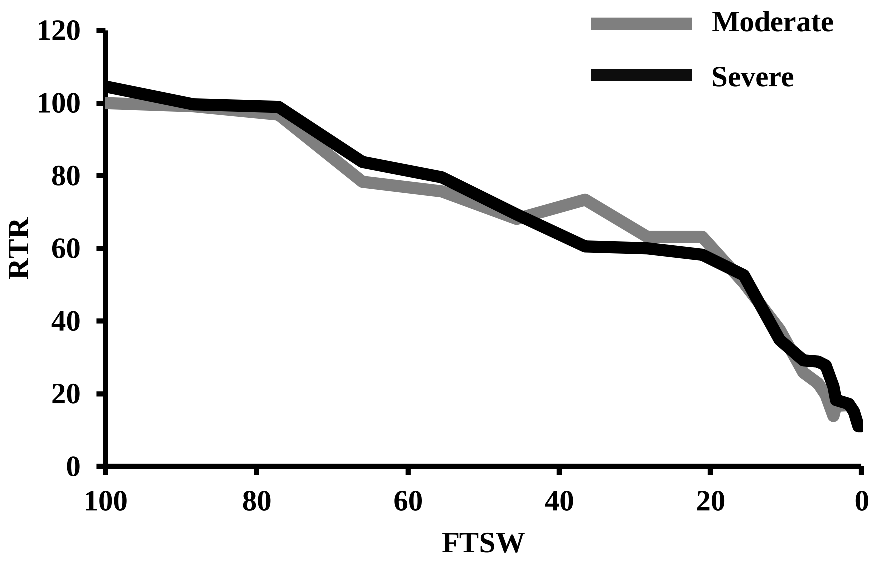

Figure S1. Changes in the relative transpiration rate (RTR) over the course of drought treatments according to the decrease of the fraction of transpirable soil water (FTSW). FTSW100 (i.e. FTSW of 100%), corresponded to the initial pot weight before applying the drought treatment. At moderate stress, FTSW12, (i.e. FTSW of 12%), RTR was 50% of that in well-water trees. At severe stress, FTSW3 (i.e. FTSW of 3%), RTR was 20% of that in well-trees.

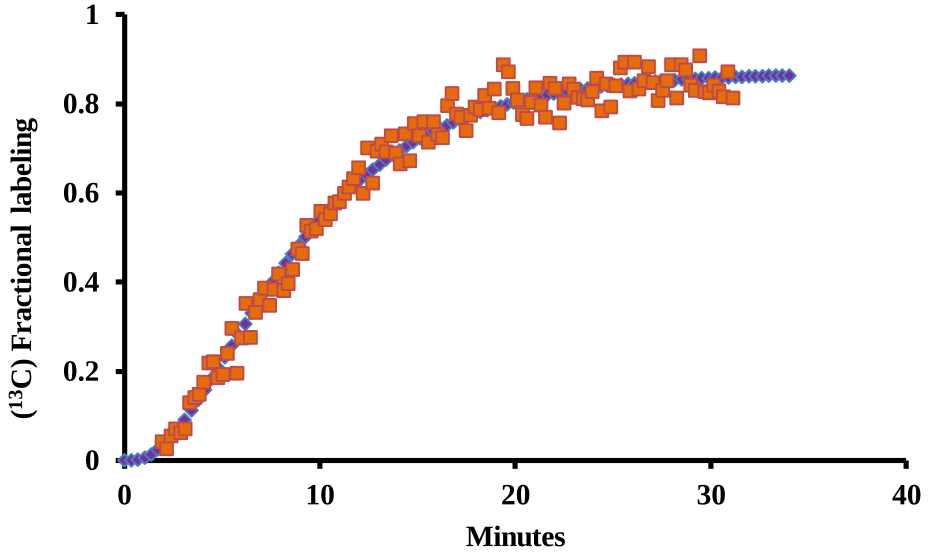

Figure S2. Representative time-course of the fractional ^{13}C-labeling of the molecular ion of isoprene after incorporation of $^{13}CO_2$ under control conditions (orange squares), and model fit to calculate the MEP pathway flux (purple squares). The fractional labeling was calculated by summing all ^{13}C atoms incorporated into the various isotopologues (see Figure S5) as described under Methods. The data were fitted to Equation (1) as described under Methods after entering the plastidial pool sizes of DXP, MEcDP and IDP+DMADP . The line represents a fitted pathway flux of 5.50 pmol mg dw^{-1} min^{-1} (see also Figure 6).

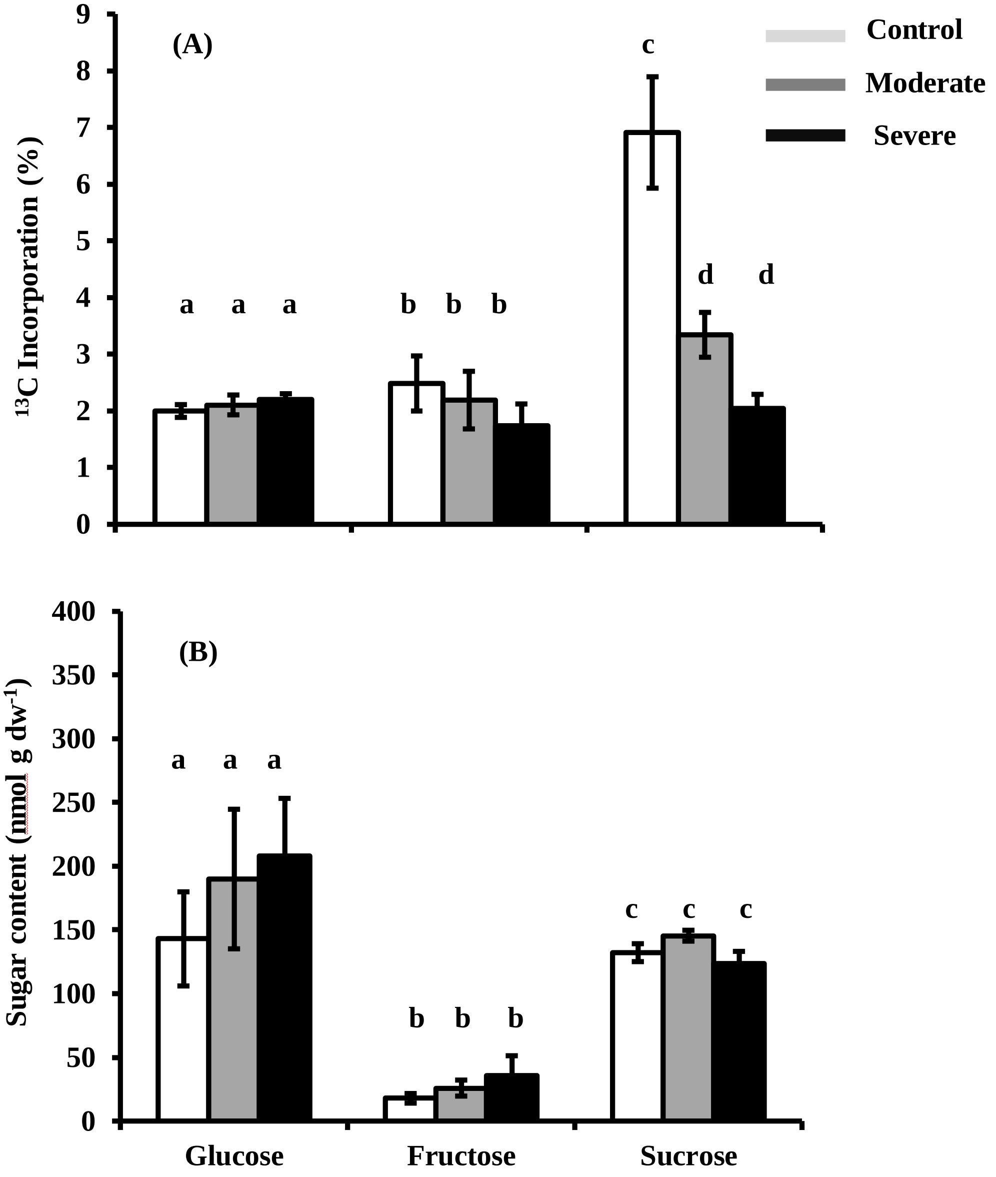

Figure S3. Effect of drought treatment on labeling of sugars from $^{13}CO_2$ and sugar content. (A) Percentage of ^{13}C incorporation in glucose, fructose and sucrose after 50 min labeling in plants growing under control, moderate and severe stress conditions. (B) Pool sizes of glucose, fructose and sucrose in needles of control, moderate and severe treatments. Data shown are means of five biological replicate ±SE. Different letters indicate significant differences at $P < 0.05$.

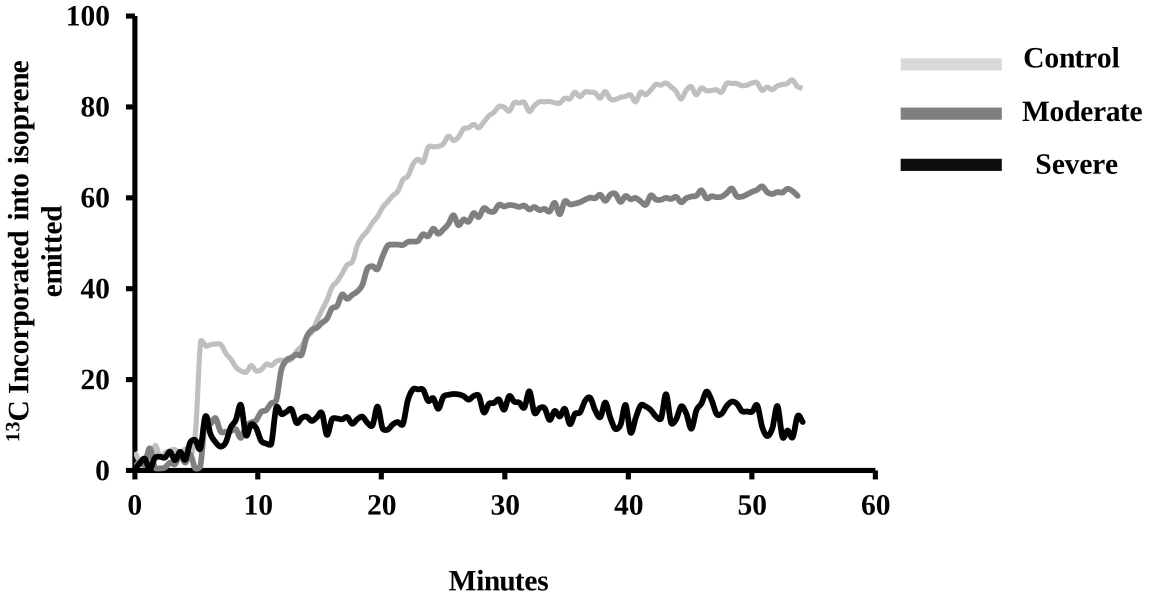

Figure S4. Percentage of ^{13}C incorporation in isoprene after labeling with $^{13}CO_2$ under control conditions, moderate and severe treatments. The figure shows a representative dataset from each of the treatment conditions.

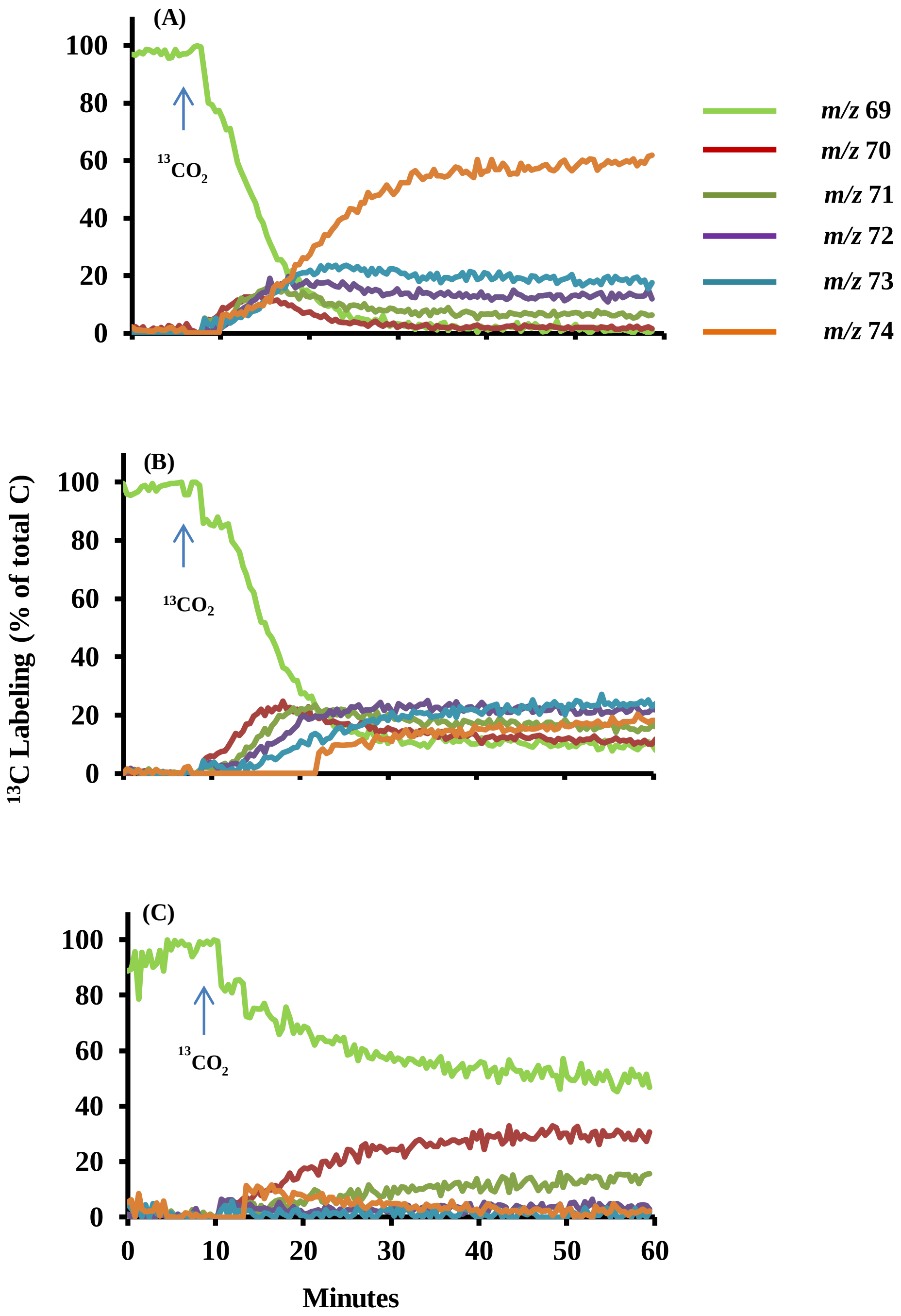

Figure S5. Representative datasets for the time-course of ^{13}C labeling of the molecular ion of isoprene emitted by P. *glauca* after incorporation of $^{13}CO_2$ under (A) control conditions, (B) moderate water stress and (C) severe water stress. Arrow indicates the time when $^{13}CO_2$ replaced the $^{12}CO_2$ in the air stream.

Supplemental Table S1. Drought did not affect the stored monoterpene content of needles. Data shown are means of five biological replicate ±SE. Different letters indicate significant differences at $P < 0.05$.

Monoterpene concentration (ng mg DW^{-1})	Control		Moderate		Severe	
	Mean	SE	Mean	SE	Mean	SE
Tricyclene	64 a	7	61 a	11	63 a	7
α-Pinene	204 b	30	193 b	40	228 b	14
Camphene	429 c	49	408 c	78	464 c	53
β-Pinene	31 d	3	25 d	5	28 d	2
Limonene	95 e	33	59 e	15	82 e	14
Camphor	160 f	35	159 f	43	205 f	35
α-Terpineol	216 g	31	199 g	50	188 g	28
Bornyl acetate	621 h	116	564 h	131	501 f	60
Sum	1820 i		1668 i		1760 i	
